# A child with cobalamin C deficiency caused by complex heterozygous variation of c.567dupT and c.80A > G complicated with pulmonary arterial hypertension and hydrocephalus: A case report and literature review

**DOI:** 10.1097/MD.0000000000048340

**Published:** 2026-05-01

**Authors:** Xuxia Cui, Yajing Zhong, Chongjuan Yin

**Affiliations:** a Pediatric Department, First Hospital of Shanxi Medical University, Jiefang Road 85, Yingze District, Taiyuan, Shanxi, People’s Republic of China.

**Keywords:** cblC deficiency, cobalamin C deficiency, diffuse lung disease, hydrocephalus, methylmalonic acidemia, pulmonary arterial hypertension

## Abstract

**Rationale:**

Cobalamin C (cblC) deficiency is one of the most common congenital vitamin B12 metabolic abnormalities, and may cause severe neurologic symptoms, gastrointestinal and nephritic symptoms.

**Patient concerns:**

A 9-month-old boy presented with a 10-day history of progressive dyspnea and weak cough, accompanied by moaning, perioral cyanosis, and poor feeding. The parents also reported significant developmental regression and delay, characterized by an inability to raise his head, sit independently, or vocalize “dada” and “mama”: milestones typically achieved by this age.

**Diagnosis:**

The patient was diagnosed with cblCdeficiency, complicated by combined methylmalonic acidemia and homocystinuria, based on clinical manifestations (developmental regression, cyanosis, pulmonary arterial hypertension (PAH)) and confirmatory genetic testing (compound heterozygous variants in the methylmalonic aciduria cobalamin deficiency type C with homocystinuria gene: c.567dupT and c.80A > G).

**Interventions:**

Following admission, the patient received multifaceted treatment. Metabolic therapy included hydroxocobalamin, folic acid, betaine, and L-carnitine to address the methylmalonic acidemia and homocystinuria. Antibiotic therapy with cefotaxime was administered for concurrent pneumonia. Additionally, bosentan (64 mg/day) was initiated for the management of PAH.

**Outcomes:**

At discharge, the patient exhibited stable vital signs, improved developmental milestones, reduced pulmonary artery systolic pressure, normal renal function, and no evidence of hydrocephalus progression. Genetic analysis revealed a genotype-phenotype correlation: the c.567dupT variant was associated with neurodevelopmental disorders and early-onset severe disease, whereas the c.80A > G variant correlated with PAH and renal dysfunction.

**Lessons:**

This report highlights the diverse clinical manifestations of cblC deficiency based on specific methylmalonic aciduria cobalamin deficiency type C with homocystinuria mutations. A review of the literature supports these genotype-phenotype associations, aiding in prognostic stratification and targeted management.

## 1. Introduction

Methylmalonic acidemia (MMA) is caused by abnormal metabolism of methylmalonyl-coenzyme A mutase (MCM) or its coenzyme cobalamin (also known as vitamin B12), which can be divided into 2 categories according to the types of enzyme defects: MCM defects and coenzyme cobalamin metabolism disorders. MMA with homocysteinemia (Hcy) may be caused by 5 genetic defects in intracellular cobalamin C (cblC) metabolism (cblC, cblD, cblF, cblJ, and cblX). CblC type combined with MMA and Hcy is the most common intracellular cobalamin pathway defect, which mainly presents some nonspecific symptoms^[1–3]^ including neurological, renal, gastrointestinal and hematological symptoms. Meanwhile, cases of severe pulmonary arterial hypertension (PAH), diffuse lung disease (DLD), hydrocephalus and kidney involvement are rarely reported, and the pathogenesis is still unclear. The results of whole genome sequencing of these children showed that the genotype was clustered in the mutant spectrum methylmalonic aciduria cobalamin deficiency type C with homocystinuria (MMACHC) gene. At present, more than 200 mutation sites of this gene have been found,^[4]^ including homozygous variation and heterozygous variation, while the complex heterozygous variation of c.567dupT and c.80A > G has been rarely reported.

Here, we present a case of child with cblC deficiency, DLD and PAH. Its prominent manifestations include MMA, hematuria, eczema, respiratory failure, PAH, and typical neurological sequelae hydrocephalus. The complex heterozygous variants of c.567dup and c.80A > G were found in MMACHC by genetic detection. The evidence for gene prediction of patients’ clinical manifestations and prognosis has been expanded.

This study has been approved by the Ethics Committee of the First Hospital of Shanxi Medical University. Written informed consent was obtained from the patient’s parents.

## 2. Case presentation

A 9-month-old boy with a history of cblC deficiency admitted to our hospital who was diagnosed on 4-month-old through genetic testing because of moderate to severe megaloblastic anemia. At the time of admission, the patient has been dyspneic for 10 days, with weak cough and gradually worsening, accompanied by moaning, cyanosis of mouth and lips, poor feeding, without fever, vomiting, diarrhea and other symptoms. Parents stated that the baby could raise his head at 3 month-old, but at 9-month-old the baby cannot raise, sit and turn over, cannot call “dad and mom”, with the developmental regression and delay.

At admission, the patient’s body temperature was 36.8°C, heart rate was 120 per min, respiratory rate was 50 per min, body weight was 7.5 kg, pulse oxygen saturation was 75%. There were lethargy, poor response, shortness of breath, positive nasal fan and inspiratory 3 depression sign, pale face, cyanosis of the lip nail bed, no jugular vein irritation in him. There were small bubbling sounds and wheezing sounds in both lungs and no cardiac murmurs on auscultation of the chest. The heart sounds were strong and consistent. No pathological murmurs or pericardial fricatives were heard in each valvular area. The liver was 5 cm below the right rib, soft in quality, and the spleen was not touched below the rib.

**Figure 1.**
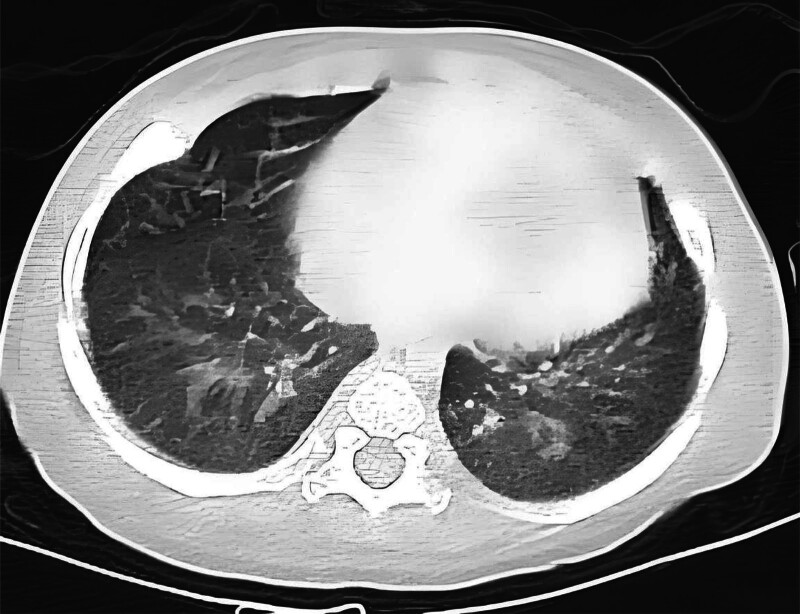
Lung HRCT showed diffuse ground-glass opacity, small amount of fluid in the interlobular pleural space. HRCT = high-resolution computed tomography.

**Figure 2.**
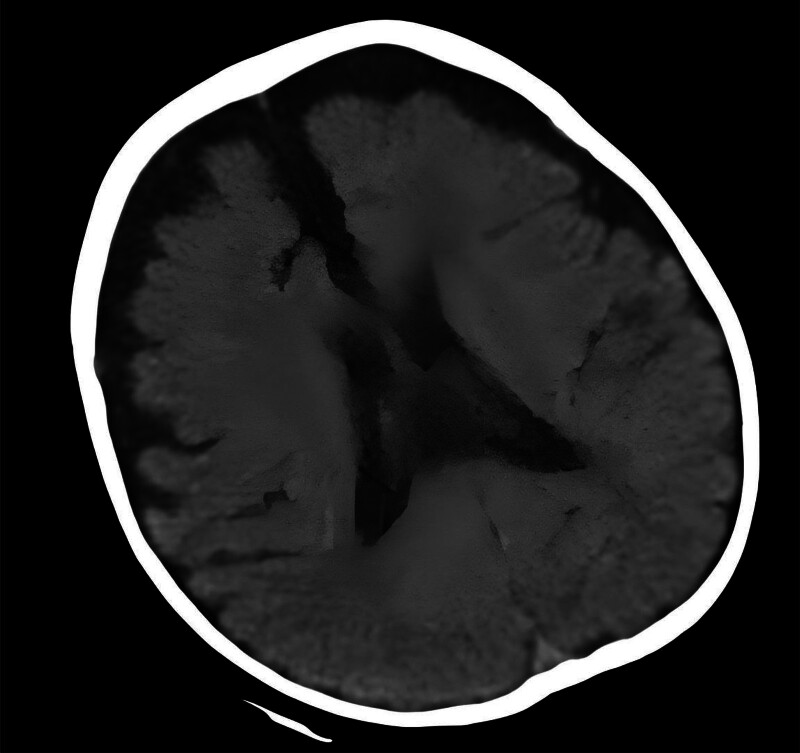
Craniocerebral HRCT showed the bilateral frontotemporal subarachnoid space was enlarged, the brain sulci and brain fissure were widened and deepened, external hydrocephalus. HRCT = high-resolution computed tomography.

**Figure 3.**
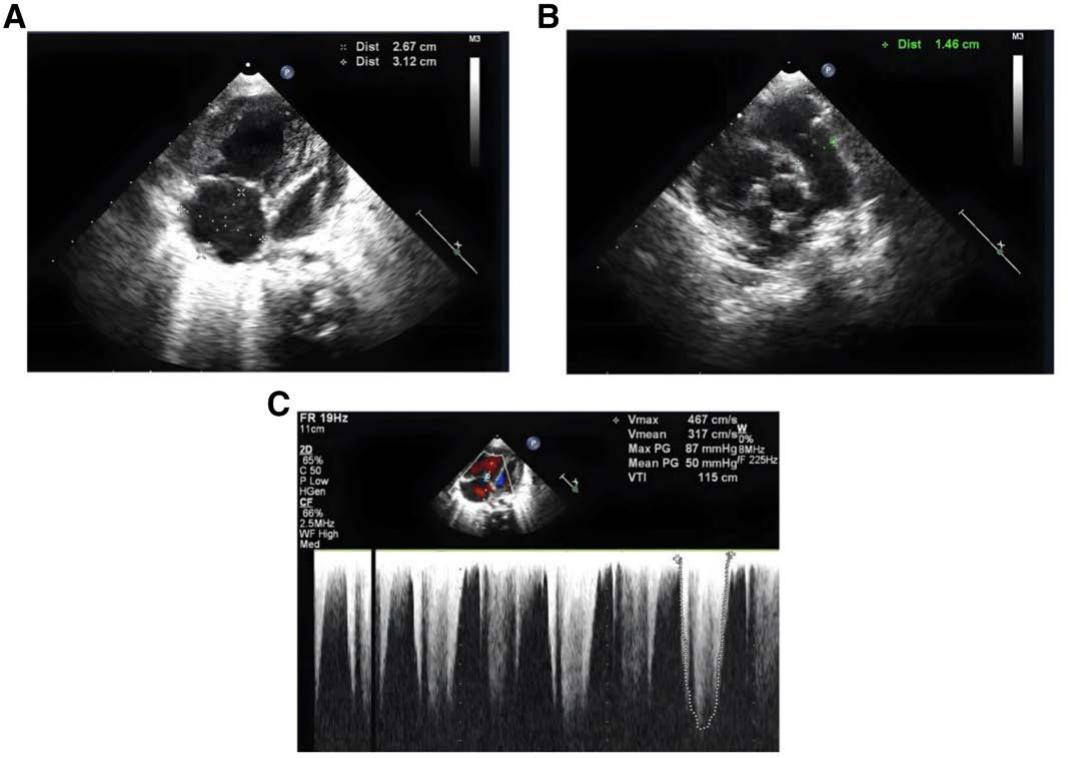
(A) The right atrium and right ventricle were enlarged, the left heart was changed under pressure, and the right ventricle wall was thickened; (B) CW Doppler evidences high-velocity tricuspid insufficiency: 4.67 cm/sec corresponding to pulmonary pressure of 87 mm Hg; (C) The inner diameter of pulmonary artery widened, 1.46 cm. C = color doppler, CF = color flow, CW = continuous wave doppler, FR = frame rate, HGen = harmonic generation, P = power doppler, PG = pressure gradient, V = velocity, VTI = velocity time integral, WF = wall filter.

**Figure 4.**
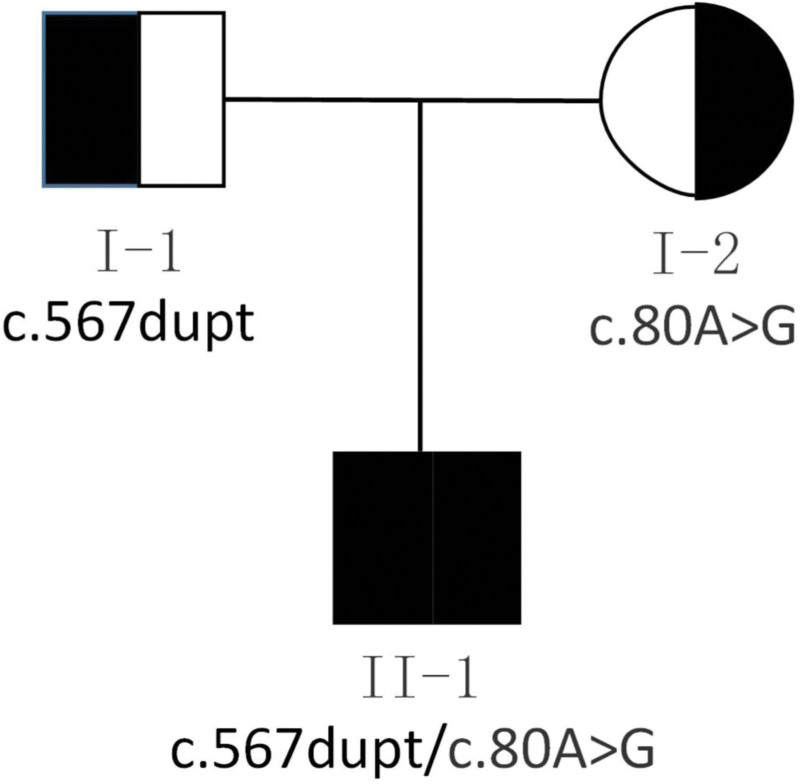
Two MMACHC mutations, c.567dupT (p.Ile190TyrfsX13) and c.80A > G (p.Gln27Arg) were found in the patient (II-1). These were inherited from paternal (I-1) and maternal (I-2) MMACHC alleles respectively. MMACHC = methylmalonic aciduria cobalamin deficiency type C with homocystinuria.

The laboratory detection showed a white blood cell count of 6.4 × 10^9^/L with 66.5% neutrophils, hemoglobin 111 g/L, increased mean corpuscular volume: 113.2 fL, mean corpuscular hemoglobin: 34.2 pg, increased mean corpuscular hemoglobin concentration:302 g/L. After treatment: red blood cell (RBC): 4.10 × 10^12^, Hb: 131 g/L, platelets: 172 × 10^9^/L, mean corpuscular volume: 108 fl, mean corpuscular hemoglobin: 32.0 pg, mean corpuscular hemoglobin concentration: 295 g/L, Blood biochemistry: total protein: 43.6 g/L, albumin: 32 g/L, globulin: 11.6 g/L, calcium: 2.08 mmol/L, potassium: 3.12 mmol/L, sodium: 124–136 mmol/L, chlorine: 91.5 mmol/L, aspartate aminotransferase: 93 U/L, bicarbonate: 18.8 mmol/L, lactate dehydrogenase: 132 U/L. The serum C-reactive protein (CRP) concentration and renal function were normal. Arterial blood gas analysis showed type I respiratory failure and a mild metabolic acidosis. The urinalysis showed urine protein ranging from negative to 1 + and RBCs from 6 to 24/hp, with 80% abnormal RBCs including mouth shape and target shape. 25-hydroxyvitamin D in serum was 54.33 nmol/L, thyroid stimulating hormone in serum was 6.37 uIU/mL, stool for routine was normal, Hcy:112.80 micromol/L, 30.1 micromol/L after 1 week of treatment. Chest Xray showed the reduced light transmittance and flaky high-density shadows in both lungs, notably in the right lung, and the heart shadow was enlarged. An high-resolution computed tomography (HRCT) scan of his chest showed diffuse ground-glass opacification in interstitial pulmonary edema, right heart enlargement, pulmonary artery widening, the pleura was not thick, and a small amount of fluid in the bilateral interlobar pleural space (Fig. 1). An HRCT scan of his head showed that the bilateral cerebral hemispheres were symmetrical, the boundary between gray and white matter was clear, and the bilateral frontotemporal subarachnoid space was enlarged, widening and deepening of cerebral sulci and fissure were observed, and external hydrocephalus was considered (Fig. 2). Cardiac ultrasound showed severe pulmonary hypertension, pulmonary artery systolic blood pressure of 89 mm Hg, right atrial and right ventricular enlargement, right ventricular wall thickening, mild tricuspid valve insufficiency, and reduced total cardiac function (Fig. 3). Genetic analysis confirmed a compound heterozygosity in MMACHC, with c.80A > G (p.Q27R) and c.567dup sequence variants, inherited from the patient’s mother and father, respectively (Fig. 4). Demographic and clinical features, laboratory features, imaging features of the patient with combined MMA and Hcy were listed in Table 1.

**Table 1:** Demographic and clinical features, laboratory features, imaging features of the patient with combined MMA and hcy.

Clinical features		Imaging findings		Demographics		Laboratory findings			
Respiratory rate	50/min	Lung Xray	Flaky high-density shadows, the heart shadow increased	Gender	Male	Hb (g/dl) MCV (fL)	111 g/L 108 fL	Kidney function, liver function	AST:93 U/L
Heart rate	130–140/min	Lung CT	Interstitial pulmonary edema, diffuse ground-glass opacity	Age	9 months	Urine protein, Urine erythrocytes	negative~+ 12–24/HP	Serous folate	52.33%
Cyanosis	positive	Brain CT	external hydrocephalus	Onset age	4 months	Arterial blood gas analysis	PaO_2_: 51 mmH_2_O mild metabolic acidosis	Complement C3,C4	increased
Fundus examination	unidentified	EF	75%	Weight	7.5 kg	Serum LDH	132 IU/L	Thyroid hormones	normal
Follow-up	6 months	PAH	87 mmH_2_O	Height	65 cm	Serum vitamin B12	725 pmol/L	Immune globulin	IgG,IgA decreased
Prognosis	improved			Head circumference	40 cm	Hcy	112.8 pmol/L	Lymphocyte subsets	normal
MMACHC gene	c.567dupt /c.80A > G					MMA concentrations	90 μmol/L	ANA,dsDNA,ACA, ANCA	normal
Genotype	cblC					Coombs test	negative	Bone marrow examination	normal

ACA = anticardiolipin antibody, ANA = antinuclear antibody, ANCA = antineutrophil cytoplasmic antibody, AST = aspartate aminotransferase, cblC = cobalamin C, CT = computed tomography, dsDNA = double-stranded DNA antibody, EF = ejection fraction, Hb = hemoglobin, Hcy = homocysteine, HP = helicobacter pylori, IgA = immunoglobulin A, IgG = immunoglobulin G, LDH = lactate dehydrogenase, MCV = mean corpuscular volume, MMA = methylmalonic academia, MMACHC = methylmalonic aciduria cobalamin deficiency type C with homocystinuria, PAH = pulmonary arterial hypertension, PaO_2_ = partial pressure of oxygen in arterial blood.

CblC deficiency was confirmed in this patient. After admission, he was treated with hydroxycobalamin (China Resources Double‐Crane Pharmaceutical Co., Ltd., Beijing, China, 1 mg intramuscular injection daily) for partially restoring the activity of MCM and reducing the production of methylmalonic acid and its toxic precursors, and restoring the activity of methionine synthase; the folic acid (Changzhou Pharmaceutical Factory Co., Ltd., Changzhou, China, 5 mg/day orally) for rapidly converting into tetrahydrofolate and other active forms to support nucleotide synthesis and cell proliferation, which is an indispensable auxiliary support part of the overall treatment plan. The betaine (Northeast Pharmaceutical Group Shenyang First Pharmaceutical Co., Ltd., Shenyang, China, 250 mg/kg orally once daily) for activating the alternative remethylation pathway to directly reduce homocysteine without relying on B12 and folic acid. The L-carnitine (Jinan Asia Pharmaceutical Co., Ltd., Jinan, China, 100 mg/kg orally once daily) for combining and discharge the accumulated organic acids to restore the coenzyme A pool, so as to directly detoxify and protect cells from damage caused by dietary and metabolic residual toxins. The cefotaxime (Youcare Pharmaceutical Group Co., Ltd., Beijing, China, 50 mg/kg, intravenous infusion, twice a day) as one of broad-spectrum antibiotics was infused for treating pneumonia. Pulmonary hypertension was revealed by pulmonary HRCT and cardiac ultrasound, and then Posentan (China Resources Double‐Crane Limin Pharmaceutical [Jinan] Co., Ltd., Jinan, China, 64 mg/day) was given. Posentan as a nonpeptide, orally effective dual endothelin receptor antagonist can antagonize both endothelin receptor type A receptors and endothelin receptor type B receptors simultaneously and block the binding of endothelin-1 to the receptor for eliminating the strong vasoconstrictive effect caused by endothelin and providing significant vasodilation thereby reducing pulmonary vascular resistance, eliminating the proliferation-promoting and antiapoptotic signals of endothelin-1 on vascular smooth muscle cells and fibroblasts, which helps to slow down or prevent abnormal thickening and fibrosis of the pulmonary vascular walls, delaying the progression of the disease.

When the patient was discharged from the hospital, the child’s vital signs were stable, with no fever, cough and cyanosis. The ill child had developmental delay but improved, he can raise his head and smile, cannot sit alone and stand. The pulmonary artery systolic pressure (PASP) was 59 mm Hg, higher than normal. The renal function was normal, RBCs in urine ranged from 5–10/hp. There was no head circumference increased or intracranial hypertension (sunset eyes, bulging anterior fontanel, vomiting) in hydrocephalus. At the 6-month follow-up (at 16 months of age), the child’s vital signs were stable, could sit alone, stand, and call “mom” and “dad”, and PASP was 35 mm Hg. The RBCs in urine ranged from 5–10/hp. No signs of hydrocephalus occured. No relevant imageological examinations were conducted.

## 3. Discussion

Initially the patient had the clinical manifestations of pneumonia such as dyspnea, cyanosis and hypoxemia, however, after the control of infectious pneumonia with antibiotic treatment for ten days, the dyspnea and cyanosis did not show significant improvement, the PAH was diagnosed through cardiopulmonary imaging tests. Although the incidence of MMA combined with pulmonary hypertension is lower, if MMA patients present with persistent cyanosis and hypoxemia, we should be vigilant about PAH. The appropriate metabolic examination and cardiopulmonary imaging tests should be conducted in time.

PAH is characterized by intra-acinar pulmonary artery occlusion, which leads to increased pulmonary vascular resistance and pressure, ultimately leading to right ventricular failure, with a 3-year mortality rate of 25% in children.^[5,6]^ The mechanism of methylmalonic acid combined with PAH may be that when methylmalonic acid combined with elevated Hcy, Hcy sulfhydryl oxidation produces a large number of superoxidation products, which promote the release of inflammatory cytokines (monocyte chemoattractant protein-1, Interleukin-8) and inhibit the production of vasoactive substance nitric oxide.^[7,8]^ Eventually, it results in vascular endothelial cell injury, intimal hyperplasia, vascular smooth muscle dysfunction, and vascular remodeling, which leads to PAH.^[9,10]^ Hcy can also increase blood viscosity and change the function of thrombolodulin, thereby reducing the antithrombotic ability of vascular endothelial cells and resulting in the formation of vascular microthrombus, and then PAH.^[11–13]^ In addition, methylmalonic acid accumulation, energy metabolism disorder and organic acid accumulation, both of which damage pulmonary blood vessels and ultimately lead to PAH.^[1]^ The coexistence of PAH associated with MMA and renal thrombotic microangiopathy suggests that the mechanism of pulmonary hypertension and renal vascular involvement may be similar. The pathological characteristics of MMA patients with pulmonary hypertension all show pulmonary veno-occlusive disease, namely, intimal fiber hyperplasia of pulmonary venules, lumen stenosis or occlusion, and interstitial pulmonary edema.^[10,14]^ On the other hand, it has also been suggested that pulmonary veno-occlusive disease is caused by pulmonary vasospasm due to significant changes in PASP.^[15]^ Another study showed that in a large prospective study of 23,437 adults recruited in the United States, higher levels of circulating MMA were significantly associated with increased all-cause mortality and cardiovascular mortality, especially in those participants with normal cobalamin.^[3]^

Three urine tests showed microscopic hematuria, indicating kidney involvement in the patient. At present, cases of renal complications caused by cblC deficiency were rarely reported. Renal involvement of cblC deficiency is usually manifested as intravascular hemolysis, hematuria, proteinuria, oliguria, hypertension, and renal insufficiency, of which hemolytic uremic syndrome is the most common.^[16]^The most common pathological manifestation of cblC-deficient nephropathy is thrombotic microangopathy, which is a serious complication of cblC deficiency. It is a typical vascular injury associated with small artery and capillary thrombosis and is found in both the kidneys and lungs of patients with atypical hemolytic uremic syndrome and PAH_,_^[4]^ an acute complication and cause of death in young patients with atypical hemolytic uremic syndrome-cblC deficiency.^[10]^ A recent study^[17]^ suggested that variant c.80A > G may be associated with prominent renal complications in Chinese patients with cblC deficiency, and megaloblastic anemia and hyperhomocysteinemia are useful clues for diagnosing hematuria and proteinuria caused by cblC deficiency. These findings are further supported by the presence of c.80A > G variants, megaloblastic anemia, and hyperhomocysteinemia in this child.

This ill child had nerve injury, which was manifested as retardation of movement, developmental retardation and decreased muscle tone. Nerve injury is a common complication in MMA patients, mainly manifested as lethargy, convulsion, psychomotor delay, ataxia, and optic nerve atrophy.^[4,18,19]^ White matter injury, myelin abnormalities, progressive brain volume loss, and cortical atrophy are the most common brain MRI findings in MMA patients. Cephalic HRCT showed hydrocephalus in this child, but there was no apparent enlargement of head circumference or intracranial hypertension (sunset eyes, bulging anterior fontanel, vomiting), possibly because the hydrocephalus was partially compensated by parenchymal atrophy. Hydrocephalus is a serious complication of MMA. Due to unclear clinical manifestations, it is often misdiagnosed as congenital malformation.^[4]^ Hydrocephalus mainly occurs in patients with early-onset cblC deficiency (95.7%), and its pathogenesis is unknown. The underlying mechanism may involve: The high concentrations of homocysteine as the angiotoxic substances may cause endothelial cell dysfunction and oxidative stress, inhibit the synthesis and activity of nitric oxide and impair vascular dilation function, promote thrombosis (hypercoagulable state) and increase the risk of microthrombi.^[20]^ As a result, leads to lesions, inflammation and thrombosis in the tiny blood vessels of the brain, affecting cerebral blood flow and venous return. The above-mentioned microvascular lesions and possible microthrombi will increase the pressure of the intracranial venous system exceeding cerebrospinal fluid pressure, that seriously affect the circulation of cerebrospinal fluid.^[21–23]^ Metabolic toxins (MMA and Hcy) and secondary inflammatory factors may disrupt the integrity of the blood-brain barrier, and impaired blood-brain barrier function may further affect cerebrospinal fluid dynamics and exacerbate the imbalance between its production and absorption.^[24]^ Secondary neuronal injury and brain atrophy, long-term accumulation of metabolic toxins leads to progressive neuronal damage, poor myelin formation and glial cell hyperplasia. This may lead to a certain degree of “cavity” effect with brain tissue atrophy, in turn cause the passive dilation of the ventricles. Currently, studies suggested that the accumulated metabolic products may have a direct toxic effect on the choroid plexus epithelial cells responsible for cerebrospinal fluid secretion or the arachnoid granulosa cells responsible for absorption, interfering with their normal functions.^[21,24,25]^

MMA was early onset in the patient, with kidney lesions, nervous system damage and hydrocephalus, severe pulmonary hypertension and pulmonary interstitial disease, as well as skin eczema, diarrhea. Studies^[26]^ have shown a high mortality rate in patients with renal manifestations (44%) and an increased mortality rate in patients with concurrent neurological disease (56%) or cardiopulmonary disease (79%). These studies suggested that the child’s condition was critical and the prognosis was poor, the mortality was higher, so we should pay close attention to monitor the condition. The incidences of ocular manifestations and visual impairments were higher in patients with cblC deficiency, affecting up to 50% of infant patients, including perimacular pigmentation, surrounded by pigmentation rings, sometimes accompanied by nystagmus, microcephaly and hydrocephalus, but the mechanism was unknown and current treatments were not effective in improving vision.^[1,2]^ The eye condition of this child did not be paid attention to, and literature review showed that the incidence of ocular involvement was quite higher.

Regarding the genetic mutations that cause the disease, a total of 286 genetic variants of the MMACHC gene were reported in the National Center for Biotechnology Information ClinVar database, 91 of which were classified as pathogenic or potentially pathogenic. The main mutation was single nucleotide (62%), followed by short repeats (19%) and deletions (18%).^[27]^ Limited representative data^[28]^ indicated that the most common mutation was c.271dupA (p.Arg91LysfsTer14), and that in homozygous states and several complex heterozygous combinations, c.271dupA was associated with early-onset disease. c.394C > T (p.Arg132Ter) was the second most common mutation. It has been reported that the most common pathogenic variants of MMACHC in Chinese population were c.482G > (36.6%), c.609G > (16.1%), c.658_660delAAG(9.8%), c.80A > G (8.0%) and c.567dupT.^[29]^ There may be differences in clinical phenotype, biochemical results, imaging findings and prognosis of different gene mutation sites. This patient’s genotype was a complex heterozygous mutation of c.80G > A and c.567dupT of MMACHC gene, c.80A > G (p.ln27ARG) produced missense mutation, and the 80th base was mutated from adenine to guanine (c.80A > G). Its encoding amino acid is mutated from glutamine to arginine (p.G 27ARG). The mutation of the c.80A > G gene locus may be related to the early onset of the disease. Studies have shown that patients with c.80A > G homozygote^[30–32]^ always have early onset, and this child also has early onset, supporting this view. In addition, the mutation of this gene locus may be associated with complicated kidney disease. In 5 cases of cblc-related kidney disease and genetic diagnosis reported in children and adolescents in China,^[33–37]^ MMACHC heterozygous variant c.80A > G was found. In another study^[17]^ of 7 cblC-deficient children with hematuria and proteinuria, 4 had the MMACHC heterozygous variant c.80A > G. Therefore, it is speculated that China’s dominant cblc-associated nephropathy may be highly correlated with the MMACHC pathogenic variant c.80A > G. In addition, c.80A > G is associated with pulmonary hypertension. In a study of 4 children with cobalamin C deficiency presenting with diffuse alveolar hemorrhage and pulmonary microangiopathy, all patients had pulmonary hypertension, and carried complex heterozygous mutations in their MMACHC (c.80a > G, c.609G > A).^[38]^ In another study, 7 patients with c.80A > G and c.609G > A complex heterozygous variants developed neuropsychiatric symptoms and pulmonary hypertension.^[29]^ In Chinese patients, c.80A > G mutations were associated with DLD.^[38]^ All the above symptoms were present in this child, which supported the above judgment.

c.567dupT mutation causes premature termination of frameshift at codon 189 and codon 202. Its variation may be associated with poor prognosis of the disease, as well as ocular lesions and intellectual impairment.^[39]^ The c.609G > A and c.567dupT variants were independent factors for poor prognosis, especially intellectual impairment. In addition, the odds ratio of the c.609G > A variant to poor prognosis was slightly higher than that of the c.567dupT variant, but the c.567dupT variant was higher than that of the c.609G > A variant in intellectual impairment. The study also found a strong link between the c.567dupT variant and eye abnormalities, however, the relevant reports were fewer and needed to be further confirmed. We did not evaluate the ocular complications in this case, which should be paid attention to in future work.

### Conclusions:

We found the correlation between PAH and respiratory distress in cblC patients, the potential association of the c.80A > G variant with renal and pulmonary vascular disease, and the availability of genetic testing for prognosis. The PAH should be focused on when the cblC children complicated with the respiratory distress. The variation of c.567dupT may be associated with neurodevelopmental disorders, early onset and poor prognosis, and c.80A > G variation may be associated with pulmonary hypertension and kidney dysfunction in this disease. Therefore, we could use the genetic testing for the efficient prediction of progression and prognosis.

## Acknowledgements

We thank the patient and his families for their kind cooperation.

## Author contributions

**Conceptualization:** Chongjuan Yin.

**Data curation:** Xuxia Cui, Yajing Zhong.

**Supervision:** Xuxia Cui, Yajing Zhong, Chongjuan Yin.

**Writing – original draft:** Xuxia Cui, Chongjuan Yin.

**Writing – review & editing:** Chongjuan Yin.
